# Anticorrosion Performance of Waterborne Coatings with Modified Nanoscale Titania under Subtropical Maritime Climate

**DOI:** 10.3390/polym16131919

**Published:** 2024-07-05

**Authors:** Yang Lyu, Weipeng Sun, Tingyou Feng, Wenge Li, Yong Jiang, Chenglin Zuo, Shuangxi Wang

**Affiliations:** 1 College of Engineering, Shantou University, Shantou 515063, China; 22ylv@stu.edu.cn; 2 Huaneng Power Company (Guangdong), Guangzhou 510000, China; 3 College of Merchant Marine, Shanghai Maritime University, Shanghai 201306, China; wgli@shmtu.edu.cn; 4 Shanghai Qixiangqingchen Coatings Technology Co., Ltd., Shanghai 201500, China

**Keywords:** waterborne coating, corrosion, subtropical maritime climate, titania nanoparticles, surface modification

## Abstract

Steel structures located in subtropical marine climates face harsh conditions such as strong sunlight and heavy rain, and they are extremely corroded. In this study, a waterborne coating with excellent corrosion resistance, hydrophobic ability, high-temperature resistance and high density was successfully prepared by using modified nanoscale titania powders and grafted polymers. The effects of three modifiers on titania nanoparticles and waterborne coatings’ properties were studied independently. The experimental results showed that the activation index of the modification employing methacryloxy silane reached 97.5%, which achieved the best modification effect at 64.4 °C for 43.3 min. The waterborne coating with nanoscale titania modified by methacryloxy silane exhibited the best hydrophobic effect, with a drop contact angle of 115.4° and excellent heat resistance of up to 317.2 °C. The application of the waterborne modified coating in steel structures under subtropical maritime climates showed that the waterborne titania coatings demonstrated excellent resistance to corrosion, high temperatures and harsh sunlight, with a maximum service life of up to five years. Economic analysis indicated that, considering a conservative three-year effective lifespan, this coating could save more than 50% in cost compared with conventional industrial coatings. Finally, the strengthening mechanism of the polymer coatings with modified nanoscale titania was analyzed.

## 1. Introduction

Owing to the subtropical maritime climate, steel structures in some coastal areas face severe corrosion challenges [1], where the atmosphere contains high concentrations of Cl^−^, NH_4_^+^, NO_3_^−^, SO_4_^2−^, Na^+^ and other corrosive ions [2]. Additionally, high temperature, high humidity, strong winds, intense sunlight and other factors aggravate the corrosion of steel structures [3]. Rusted surfaces contain large amounts of coal ash and high salt concentrations, making rust removal difficult. In recent years, owing to the increase in the concentration of acidic and basic oxides in the atmosphere, it has become difficult for traditional heavy anticorrosive coatings, such as zinc-rich coatings, to adapt to the current harsh corrosion environment, which often encounters widespread corrosion issues within a year of application and requires regular maintenance.

In the past few years, nanoscale titania (TiO_2_) has attracted interest as a filler of waterborne anticorrosive coatings. Its role is not only to cover and decorate the substrate, but its more important role is also to improve the physical and chemical properties of the coating: the chemical stability, corrosion resistance, ultraviolet light resistance and weather resistance of the coating. Hence, nanoscale titania can prevent cracks and moisture, thus delaying aging and extending the life of the coating. However, nanoscale titania has a high tendency of agglomeration, which can affect the properties of polymeric nanocomposite materials [4]. Moreover, the binding force between the waterborne titania coating and the substrate is not strong enough, so it is easy for it to fall off.

In recent years, researchers worldwide have explored various types of waterborne modified titania anticorrosive coatings. For example, Singh et al. [5] used acetoxime-modified titania to prepare a type of waterborne coating. Comparing the bare sample with the coated sample, it showed that the thin modified titania waterborne coating improved the vibration and noise damping of steel. Hou et al. [6] used ethylene glycol diglycyl ether (GDE) to modify titania first. Then, they mixed TiO_2_@GDE with dodecanoic acid (DA), perfluorodecyl triethoxysilane (PFTS) and GDE to prepare a super hydrophobic coating. The results showed that the water contact angle (WCA) of this modified coating was 164 ± 3 degrees. Liang et al. [7] used silane coupling agent KH560 to modify titania nanoparticles and blended them with epoxy resin E51 (EP). Then, they sprayed the mixed composite material on the substrate to form a TiO_2_/EP superhydrophobic coating with excellent mechanical and chemical stability. By adjusting the mass ratio of TiO_2_ to EP, it was found that the 1:3 TiO_2_/EP coating had excellent mechanical and chemical stability while having a contact angle of 159.5 degrees ± 1.5 degrees.

Although these types of waterborne modified titania coatings can improve corrosion resistance, they are costly and have not been widely tested in large-scale applications under harsh corrosive conditions, such as coastal power plants under subtropical maritime climate. Consequently, economically and environmentally friendly heavy anticorrosive coatings suitable for the long-term protection of coastal steel structures have become an important research focus [8].

In this study, a waterborne coating with excellent corrosion resistance, hydrophobic ability, high-temperature resistance and high density was successfully prepared by adding modified nano-titania ceramic powders in the polymers. The effects of three kinds of modifiers on titania nanoparticles and waterborne coatings’ properties were studied. And the contact angle measurement, thermogravimetric analysis and corrosion tests were carried out. The modification and formation mechanism of the coatings were analyzed. The application results of the waterborne modified coating exposed to harsh environments showed that the coating demonstrated excellent resistance to chemical corrosion, high temperatures and harsh sunlight, with a maximum service life of up to 5 years. Economic analysis indicated that this coating could save more than 50% in cost compared with conventional industrial coatings.

## 2. Materials and Experiments

### 2.1. Materials

#### 2.1.1. Materials for Surface Modification of Nanoscale Titania

Raw materials for the preparation of modified titania nanoparticles included commercially sourced high-purity powder and chemical reagents, such as rutile titania powder (100 nm) (AR, Hangzhou Hengna New Material Co., Ltd., Hangzhou, China), anhydrous ethanol, acetic acid, deionized water (AR, Shanghai Aladdin Biochemical Technology Co., Ltd., Shanghai, China) and silane coupling agents (γ-amino propyl triethoxy silane, γ-(2,3-epoxy propoxy) propyl trimethoxy silane and γ-methacryloxy propyl trimethoxy silane) (AR, Hangzhou Jessica Chemicals Co., Ltd., Hangzhou, China).

#### 2.1.2. Materials for Waterborne Coating Preparation

Polyacrylate waterborne emulsion (Joncryl^®^ 8280) (AR, BASF SE, Ludwigshafen, Germany), dispersant (PAA-NH_4_), wetting agent (alkylphenol ethoxylates) (AR, Sinopharm Chemical Reagent Co., Ltd., Shanghai, China) and defoaming agent (B-290) (AR, Datian Chemical Co., Ltd., Foshan, China) were used as raw materials to prepare waterborne anticorrosive coatings.

### 2.2. Experiments

#### 2.2.1. Surface Modification of Nanoscale Titania

**Figure 1:**
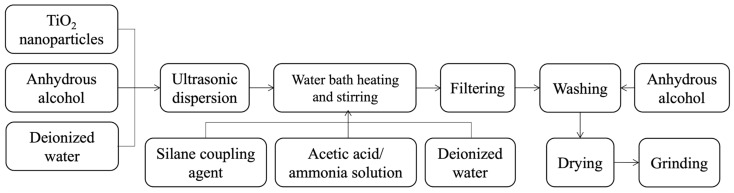
A schematic of the modification process.

Titania nanoparticles were mixed with anhydrous ethanol and deionized water (3:1 volume ratio) and dispersed by an ultrasonic dispersion processor (SM-1000C, Shunma Tech., Nanjing, China) for 10 min to prepare a titania suspension. Silane coupling agents (γ-amino propyl triethoxy silane, γ-(2,3-epoxy propoxy) propyl trimethoxy silane and γ-methacryloxy propyl trimethoxy silane) were then mixed with deionized water (10:1 mass ratio) and adjusted to a pH of 2 for hydrolysis by adding acetic acid as the catalyst. The titania suspension and the modifier solution were then mixed and heated in a constant-temperature water bath (NLDC, Jiangsu Nele Instrument Equipment Manufacturing Co., Ltd., Nanjing, China) at 40–100 °C with constant stirring at 500 r/min for 10–70 min. After that, the obtained suspension was centrifuged by a centrifugal machine (MC-15, Xiangxin Instrument, Changsha, China) for 30 min. The precipitate was separated and rinsed with anhydrous ethanol to remove excess silane coupling agent that was physically adsorbed on the surface of the titania nanoparticles. Finally, three kinds of silane coupling agent-modified titania nanoparticles (A1, A2 and A3) were obtained by drying the precipitate at 60 °C for 6 h in an electrothermal blowing dry box (GN-25A, Galainer, Suzhou, China) and grinding the dry powder into fine particles. The sample number of three kinds of modified nanoscale titania is shown in Table 1, and the modification process is illustrated in Figure 1.

#### 2.2.2. Preparation of Waterborne Anticorrosive Coatings

At room temperature, 20 g nanoscale titania powders (A1, A2, A3 and unmodified), 70 g waterborne polyacrylic emulsion and 4 g dispersant (PAA-NH_4_) were added to the ball mill tank, the volume of which did not exceed 1/3 of the ball mill tank. Then, 120 g zirconia grinding balls of different diameters (2 mm, 4 mm, 8 mm and 16 mm) were added, and ball milling (DQM2-4L, Chunlong Experimental Instrument Co., Ltd., Lianyungang, China) was carried out at a speed of 130 r/min for 24 h. The suspension after ball grinding was filtered out by a 100-mesh filter under atmospheric pressure. A total of 3 g of defoaming agent (B-290) and 3 g wetting agent (alkylphenol ethoxylates) were added to the filtrate and heated to 55 °C in the water bath. Then, it was defoamed in a vacuum defoaming machine (TP-2, Sun-tec Co., Ltd., Beijing, China) for 10 min and stirred at a speed of 500 r/min. Finally, four kinds of titania waterborne anticorrosive coatings (B1, B2, B3 and B4) were obtained, as shown in Table 2. The Q235 steel sheet surface underwent sandblasting to achieve a Sa 2.5 finish, followed by thorough cleaning with acetone to eliminate grease and other contaminants. Subsequently, the nanoscale titania waterborne anticorrosive coatings (B1, B2, B3 and B4) were painted for 80 μm thickness on the Q235 steel plate using an air spraying method at 0.5 MPa and room temperature. The coated steel plates were left to air-dry at room temperature for five days to yield the final coating samples.

#### 2.2.3. Characterizations

(1) Field-emission scanning electron microscopy (FE-SEM).

The microstructures of the unmodified and modified nanoscale titania powders (A1, A2 and A3) were characterized using a field-emission scanning electron microscope (Gemini SEM300, Zeiss, Oberkochen, Germany).

(2) Activation index

A suitable quantity of modified titania nanoparticles (A1, A2 and A3) and deionized water were mixed in a beaker, stirred for 5 min and then allowed to stand for 4 h. The precipitated powder at the bottom of the beaker was separated and dried in the electrothermal blowing dry box. The activation index of the modified powder was calculated using Equation (1).

(1) $$H=\frac{m_{1}-m_{2}}{m_{2}}\times 100\%$$

where *H* is the activation index, *m*_1_ is the total mass of the sample, and *m*_2_ is the mass of the precipitated portion of the sample. The activation index values are within the range of 0 to 1. A higher activation index indicates a more favorable modification effect [9].

(3) Contact angle (CA) tests

The water drop contact angles of the four kinds of nanoscale titania waterborne coatings (B1, B2, B3 and B4) were measured using an optical contact angle measuring device (OCA Pro15, Dataphysics, Filderstadt, Germany) to assess their hydrophobic properties.

(4) Thermogravimetric analysis (TGA)

The thermal decomposition behavior of the four kinds of nanoscale titania waterborne coatings (B1, B2, B3 and B4) was studied by a thermal gravimetric analyzer (HTG-4, Henven, Beijing, China) at a warming rate of 10 °C/min under nitrogen flow at 0–600 °C.

#### 2.2.4. Corrosion Tests

(1) Neutral salt spray

A silane methacryloxy-modified nanoscale titania waterborne coating (B3) was tested in a neutral salt spray using a brine spray test chamber (Q-FOG, Q-LAB, Cleveland, OH, USA). The temperature in the spray room was set to 35 °C, the NaCl solution concentration was 5%, the temperature in the pressure barrel was 47 °C, the input power of the air compressor was 0.4 MPa, the pressure power was 0.2 MPa and the spray power was 0.1 MPa.

(2) Coupon corrosion test

The aforementioned coating (B3) was also tested on coupons suspended in different locations of a coastal thermal power plant in China, including a boiler, an open drain, a water treatment station and a coal bunker. The size of the coating samples was 150 mm × 70 mm, with X scratches and no scratches, and they were kept at a distance of 50 mm to ensure that they were completely exposed to a corrosive environment and sunlight.

#### 2.2.5. Applications under Subtropical Marine Climate

To comprehensively assess the anticorrosion protection efficacy of the modified nanoscale titania waterborne coating (B3) under subtropical maritime climate, three typical steel structures with severe corrosion in a coastal power plant (spring hangers, boiler connecting bolts and feeder platform legs) were selected for practical engineering applications. The coatings were observed regularly for 5 years.

## 3. Results and Discussion

### 3.1. Modification Mechanism of Titania Nanoparticles

Numerous hydroxyl (–OH) groups are distributed across the surface of the titania nanoparticles, imparting them with a hydrophilic nature. The silane coupling agent (X-Si-OR), which possesses both hydrophobic (X) and hydrophilic (R) functional groups, plays a vital role in establishing stable chemical bonds between organic and inorganic materials [10]. This coupling agent incorporates hydrolyzable (R) groups that serve as intermediates in the generation of hydrophilic groups for bonding to inorganic or nanoparticle surfaces [11].

**Figure 2:**
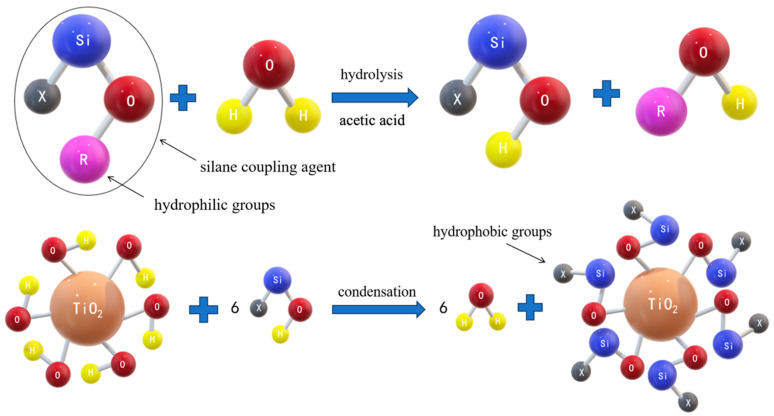
Modification mechanism of titania nanoparticles by silane coupling agent.

During hydrolysis, the alkoxy groups of the coupling agent reacted with the water in the solution and transformed into silanol groups (Si-OH), as illustrated in Figure 2. Subsequently, the surface of the nanoparticles underwent modification through a condensation reaction involving Si-OH and –OH groups on the nanoparticle surface. As a result, numerous hydrophobic groups emerged on the surface of titania nanoparticles after modification, leading to a shift from hydrophilic to hydrophobic characteristics.

As Figure 2 shows, the side chains of the modified spherical nanoscale titania extend outwards, with the resin molecules grafted onto the spherical surface, resulting in the formation of a fluffy sphere characterized by numerous side chains. This configuration is referred to as a highly branched structure [12]. Moreover, traditional linear polyesters often have strong water sensitivity mainly because of the easy hydrolysis of the ester group in the molecular chain, which is easily exposed to air. The highly branched structure of the hyperbranched polyester can form the inclusion of ester groups in the molecular chain, which effectively prevents direct contact between the ester groups and water in the air, thus reducing the probability of ester group hydrolysis.

### 3.2. Micromorphology of Titania Nanoparticles

**Figure 3:**
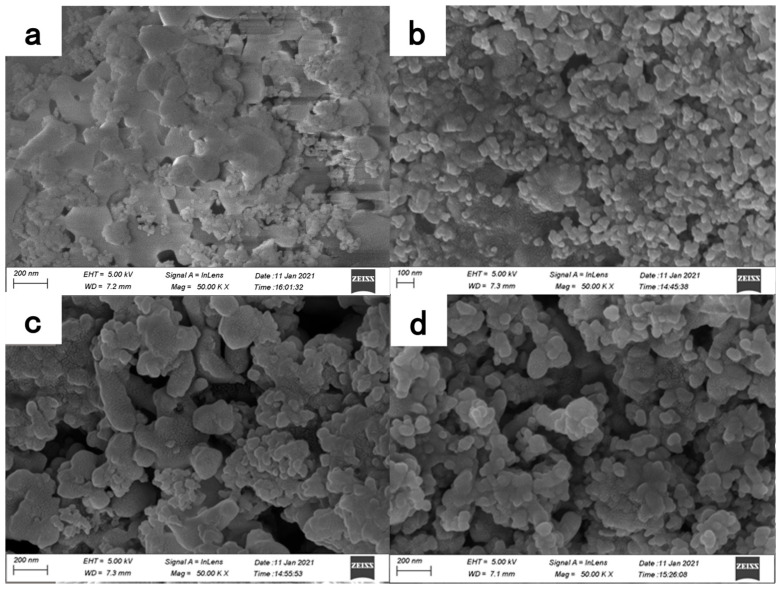
Morphology of titania nanoparticles: (**a**) unmodified; (**b**) A1; (**c**) A2 and (**d**) A3.

The micromorphologies of the titania nanoparticles (30,000× magnification) before and after modification are depicted in Figure 3. As shown in Figure 3a, the unmodified titania nanoparticles displayed severe aggregation with inconspicuous boundaries between the particles. The polar and hydrophilic nature of their surfaces leads to a spontaneous reagglomeration of micron-sized particles when dried in air [13].

However, the chemical interaction between the modifier and the surface hydroxyl groups introduces substantial steric hindrance on the modified surface of the titania nanoparticles. This hindrance plays a crucial role in impeding the agglomeration of particles during the drying of the coatings. As a result, the titania nanoparticles after surface modification displayed clear particle boundaries, and the extent of agglomeration was significantly diminished, as depicted in Figure 3b–d.

### 3.3. Activation Index of Modified Titania Nanoparticles

**Figure 4:**
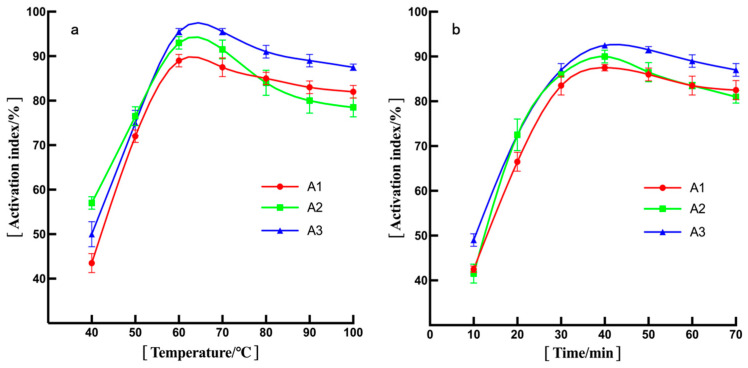
Relationship between activation index of modified titania nanoparticles and (**a**) temperature and (**b**) time.

The correlation between the modification temperature and the effectiveness of the titania nanoparticle modification is illustrated in Figure 4a. Notably, the activation index of titania nanoparticles modified by all three agents initially rises with increasing modification temperature, reaching a peak between 60 °C and 70 °C and subsequently decreasing. The modifier methacryloxy silane exhibited the most favorable modification effect at 64.4 °C, achieving the highest activation index for titania nanoparticles (A3), which amounted to 97.5%.

To explore the influence of modification time, titania nanoparticles were modified at 60 °C with an equivalent amount of three silane coupling agents for varying durations. Figure 4b illustrates the changes in the activation index of the titania nanoparticles modified using the three agents as the modification time increased. Evidently, the modifier methacryloxy silane (A3) also demonstrated an optimal modification effect, which had the highest activation index for titania nanoparticles in the three kinds of modifiers (A1, A2 and A3).

### 3.4. Self-Cleaning Property of Titania Waterborne Coatings

**Figure 5:**
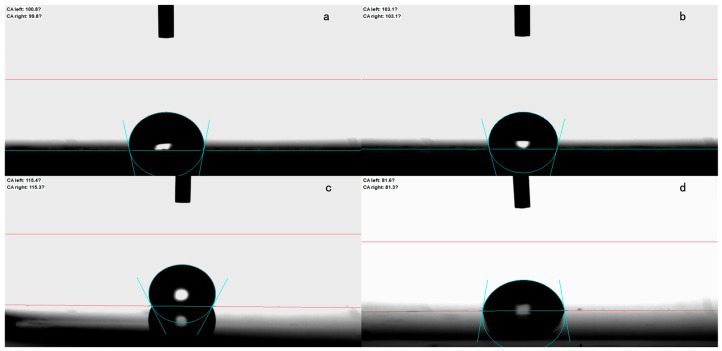
Water droplets on the surface of different waterborne coatings: (**a**) B1; (**b**) B2; (**c**) B3 and (**d**) B4.

In general, a hydrophobic surface is characterized by a water contact angle exceeding 90°, endowing it with a self-cleaning capability [14]. As shown in Figure 5a–d, the water contact angles of the coatings are 100.8°, 103.1°, 115.4° and 81.6°, respectively. The unmodified nanoscale titania waterborne coating showed poor water resistance. However, the use of the modifier methacryloxy silane proved to be a superior choice, exhibiting an outstanding waterproofing effect on the modified nanoscale titania waterborne coating (B3). The hydrophobic nature of the coating enables its self-cleaning ability for steel structures, particularly in challenging corrosive environments.

Effectively, the modified nanoparticles assembled on the coating surface, creating a hydrophobic surface that promoted the effortless rolling off of water droplets. Additionally, these rolling water droplets gather corrosive substances and other pollutants that adhere to the coating surface before eventually detaching and falling off as collective unit [15]. This mechanism enhances the protective capabilities of the coating against harsh environmental conditions.

### 3.5. Thermogravimetric Analysis of Titania Waterborne Coatings

**Figure 6:**
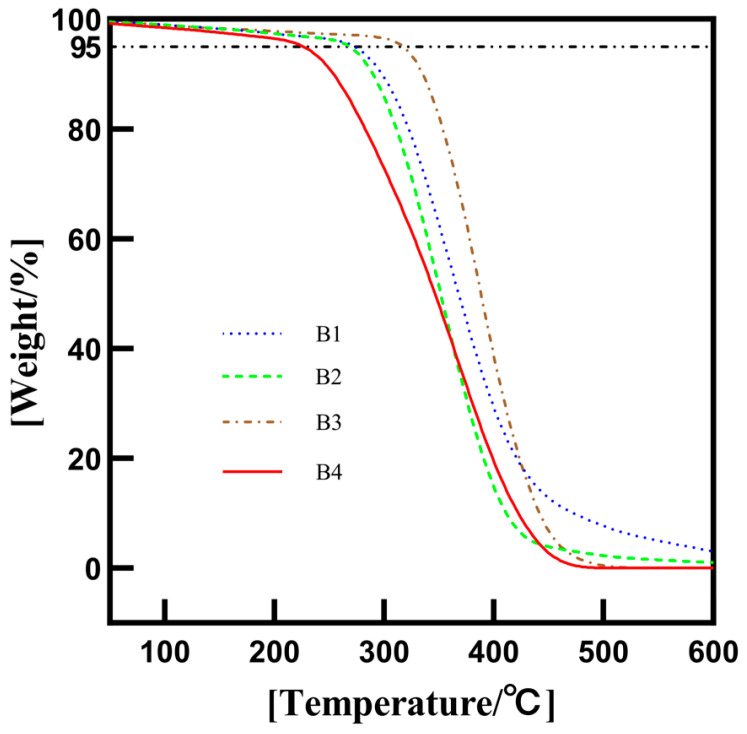
TGA curves of different waterborne nanoscale titania coatings.

The thermal decomposition behaviors of the different waterborne nanoscale titania coatings were studied using thermogravimetric analysis. The sample was heated at 10 °C/min under nitrogen flow, and the TGA curves are shown in Figure 6. It can be seen that the quality of waterborne coatings (B1, B2, B3 and B4) starts to decrease obviously at 273.3 °C, 268.1 °C, 317.2 °C and 225.6 °C, respectively. Therefore, methacryloxy silane-modified nanoscale titania is most suitable for high-temperature-resistant coatings. This phenomenon is attributed to the bonding of modified nanoscale titania with waterborne solvent molecules in the coating. The silane coupling agent binds the titania nanoparticles and the acrylic molecules together by chemical bonds so that they can be dispersed more evenly in the coating. The molecules in the coating form an infinite number of connected heat conduction channels that, when heated locally, could quickly transfer heat to other parts of the coating. Therefore, modified titania nanoparticles in the coating could improve the heat resistance of the waterborne coating [16].

### 3.6. Corrosion Performance of Methacryloxy Silane-Modified Titania Coating

#### 3.6.1. Neutral Salt Spray

**Figure 7:**
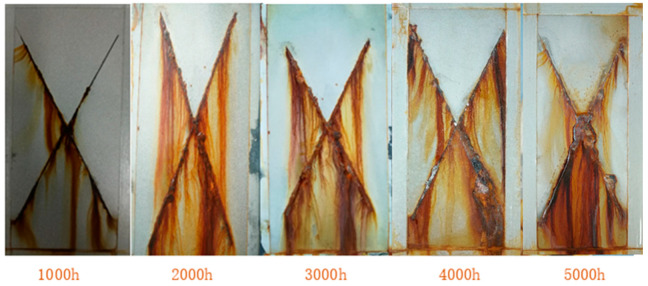
Effect of salt spray resistance test time on surface morphology of coating.

As Figure 7 shows, when the test was conducted for 2000 h, there were obvious corrosion marks on the line, and expansion corrosion was extremely weak. In the test for 3000 h, weak corrosion occurred at the scratch of the test plate, but no defects were produced on the surface of the coating. The amount of unilateral corrosion was less than 1 mm after stripping the coating in the expansion area, and there was no blistering, rust, peeling, cracking or other conditions on the surface of the non-scratch area. When the test was performed for 4000 h, the corrosion expansion at the marking area was more obvious, and the metal gloss on the surface of the test plate was slightly reduced; however, there was still no pitting, foaming, pinholes, or other defects. After stripping the coating in the corrosion expansion area, the degree of single-side corrosion expansion was approximately 1.6 mm (less than 2 mm), indicating that the silane methacryloxy-modified nanoscale titania waterborne coating could still maintain its basic anticorrosion function. In the salt spray test, the modified titania nanoparticles in the coating were constantly consumed, resulting in a decrease in the density of the coating and defects such as pinholes and bubbles inside the coating; corrosive media such as oxygen and water vapor in the air can penetrate the substrate, causing the corrosion of the substrate. When the test reached 5000 h, the unilateral expansion of the coating exceeded 2 mm, the corrosion degree of the substrate was severe and slight foaming and pinholes appeared, indicating that the coating could no longer maintain the anticorrosion function and could be considered as a failure. In conclusion, this modified nanoscale titania waterborne coating has excellent salt spray resistance and meets the design requirements for a lifetime of more than 5 years.

#### 3.6.2. Coupon Corrosion Test

**Figure 8:**
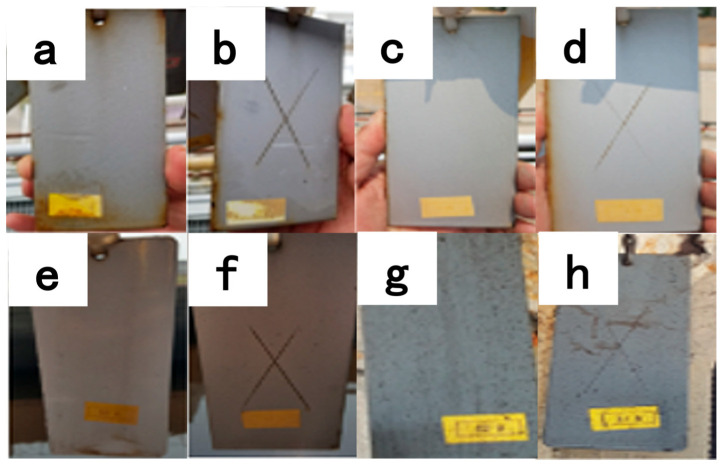
One-year coupon corrosion tests in different places: (**a**,**b**) a boiler; (**c**,**d**) open drain; (**e**,**f**) water treatment station and (**g**,**h**) coal bunker.

As Figure 8 shows, after the one-year coupon corrosion test, although a small number of pollutants accumulated on the surface of the test plates in all four areas, almost no visible corrosion defects occurred. Based on the service performance of the hanging board, this silane methacryloxy-modified nanoscale titania waterborne coating showed excellent anticorrosion effects in various typical corrosion areas, particularly in highly polluted environments; the paint film still has excellent anticorrosion performance. The coupon corrosion test achieved excellent results and can be used for further on-site tests.

### 3.7. Strengthening Mechanism of Modified Nanoscale Titania Coatings

Coatings adhere to the substrate or other coatings through adhesion and cohesive forces within the coating material, forming a physical barrier that protects the substrate [17]. However, owing to external environmental influences during the alternating process of hot and cold cycles, the thermal expansion and contraction coefficients of the coating and substrate may differ, resulting in stress differences. In addition, airflow, vibration, ultrasound or subsonic oscillations can cause significant differences in the elastic modulus between the coating film and the substrate, leading to local stress and causing the coating to peel off, thus failing to provide protection.

**Figure 9:**
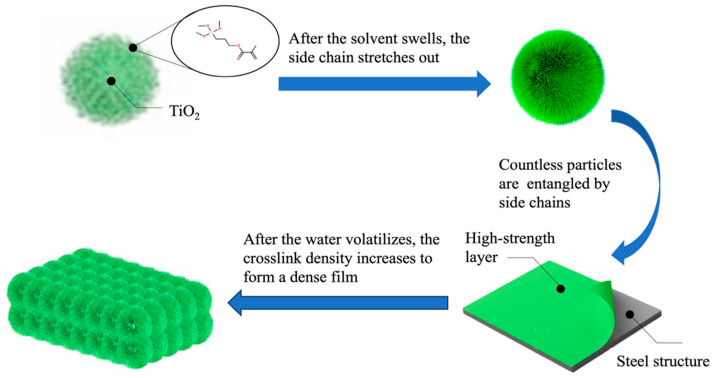
Strengthening mechanism of modified nanoscale titania coatings.

The mechanism by which the modified spherical titania nanoparticles formed a high-strength resin coating is illustrated in Figure 9. The side chains of the modified nano-titania extend, and the resin molecules are grafted onto the surface of the spherical particles, forming fluffy particles with numerous side chains [18]. Countless particles were intertwined by side chains, forming a dense and corrosion-resistant coating. The three-dimensional network structure formed by the ultrahigh molecular weight rapidly disperses the local stress between the networks, greatly reducing the damage to the coating caused by the stress. The titania nanoparticle connected one end of the silane coupling agent molecule, whose other end connected the acrylic molecule in the waterborne solvent. Therefore, a large number of long side chains on the titania nanoparticle formed, which exhibited good elasticity. These free elastic side chains ensured the full flexibility of the coating, greatly extending the protection life of the film.

Moreover, these side chains are tightly connected to the impurities, preventing the formation of gaps or cracks between the impurities and resin film owing to shrinkage during drying and solidification, thereby preventing pitting corrosion. Adjacent modified TiO_2_ particles can be reconnected owing to the presence of side chains, effectively repairing the already formed cracks [19]. This is primarily manifested when the coating is locally damaged, forming a linear wound and scar at the wound site, which helps form a dense protective layer at the scar site and prevents the further corrosion of the substrate.

### 3.8. On-Site Application of Modified Titania Coatings under Subtropical Marine Climate

#### 3.8.1. Surface Coatings on Spring Hangers at Boilers

**Figure 10:**
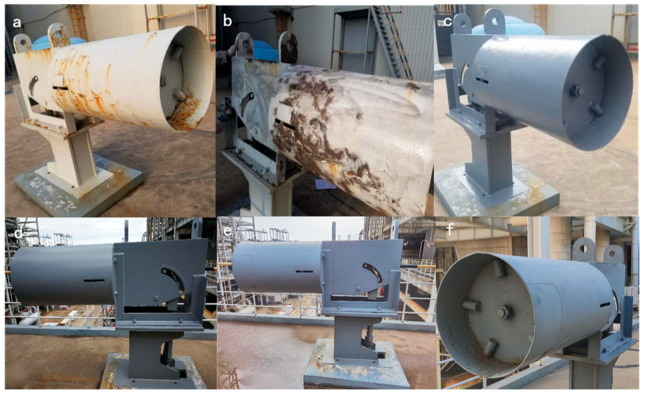
Coatings above spring hangers: (**a**) original condition; (**b**) after rust removal; (**c**) until 2018; (**d**) until 2021; (**e**) until 2022 and (**f**) until 2023.

Spring hangers are installed in environments with high humidity, strong sea breezes and sun exposure, where the air contains many chloride ions and sulfides. Hence, the surfaces of the spring hangers were severely eroded, as shown in Figure 10a.

Before coating, the steel surface was treated for rust removal, as shown in Figure 10b. A modified titania waterborne anticorrosive coating was painted in December 2018, as shown in Figure 10c. The coating surface conditions for 2021 and 2022 are shown in Figure 10d and e, respectively.

After five years of operation, in December 2023, the coating surface remained clean and smooth with no bubbling, rusting or peeling, as shown in Figure 10f.

#### 3.8.2. Surface Coatings on Boiler Connecting Bolts

**Figure 11:**
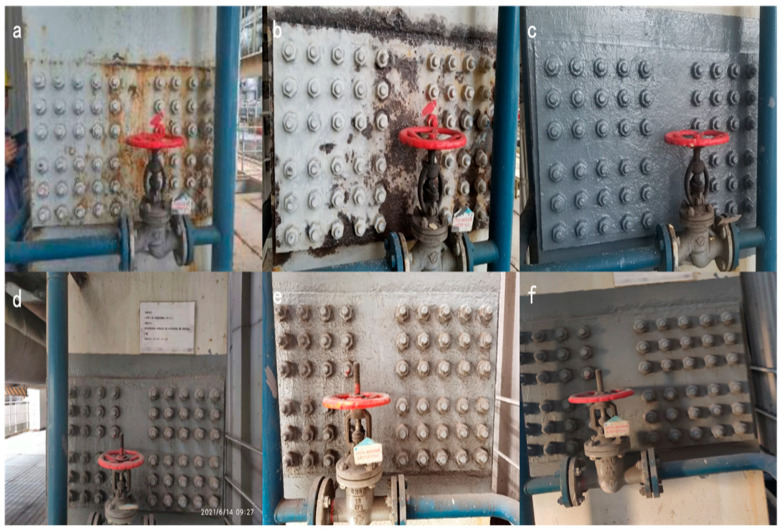
Coatings above boiler connecting bolts: (**a**) original condition; (**b**) after rust removal; (**c**) until 2018; (**d**) until 2021; (**e**) until 2022 and (**f**) until 2023.

The temperature at the boiler bolt connection is high, usually above 200 °C, and the ordinary coating easily ages and fails, as shown in Figure 11a. The modified titania waterborne coating was applied to the joints of boiler bolts to verify its anticorrosion effect in high-temperature areas.

Prior to coating, the steel surface was treated for rust removal, as shown in Figure 11b. A modified titania waterborne anticorrosive coating was painted in December 2018, as shown in Figure 11c. The coating surface conditions for 2021 and 2022 are shown in Figure 11d and e, respectively.

In December 2023, after five years of on-site testing, the coating surface remained intact without evident corrosion or peeling. The rusted screws remain intact without further corrosion. Only a small amount of rusting occurred at the edges and corners, where it was difficult to coat without significant peeling, as shown in Figure 11f.

#### 3.8.3. Surface Coatings on Coal Powders’ Feeder Platform Legs

**Figure 12:**
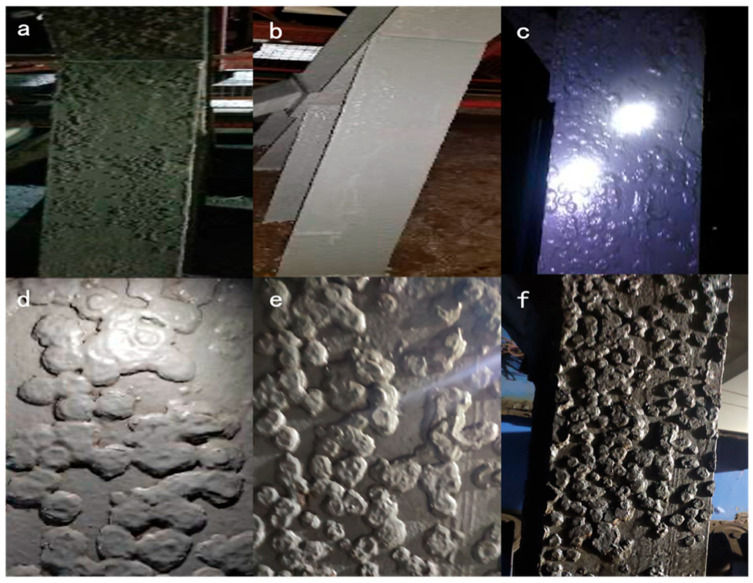
Coatings above feeder platform legs: (**a**) original condition; (**b**) until 2018; (**c**) until 2020; (**d**) until 2021; (**e**) until 2022 and (**f**) until 2023.

The feeder platform legs are located in a dark, high-humidity, high-corrosion and high-coal dust working environment; therefore, they were subjected to severe corrosion, and the surface was rusty, as shown in Figure 12a.

Before coating, the surface of the feeder platform leg was not de-rusted to observe the effect of the modified titania waterborne coating under low-grade treatment conditions. A modified titania waterborne anticorrosive coating was painted in December 2018, as shown in Figure 12b. The coating surface conditions for 2020, 2021 and 2022 are shown in Figure 12c, d and e, respectively.

After five years of on-site testing in December 2023, the coating did not exhibit obvious corrosion or peeling, as shown in Figure 12f. The coating has been successfully applied to low-grade treated steel structures, indicating that the waterborne nanoscale titania coating can significantly inhibit the further corrosion of steel structures.

In addition to the above experiments, environmentally friendly waterborne nanoscale titania coatings were applied to typical areas of this coastal power plant, including high-altitude steel structures and circular coal yard water guns, with an application area of approximately 60,000 square meters. After five years of on-site testing, the coatings remained intact with no cracking, rust spreading or other issues. These experimental cases indicate that waterborne nanoscale titania coatings can effectively solve the corrosion problems of steel structures in heavily polluted environments and under high-temperature conditions in subtropical marine environments.

### 3.9. Economic Analysis

Compared with regular oil-based coatings, the unit cost of the new water-based anticorrosion coating appears to be nearly three times higher. However, the new water-based anticorrosion coating showed a significant reduction in the required coating thickness compared with regular oil-based coatings. In other words, for the same quantity of coating, the theoretical coating area of the product in this study was two or three times that of regular oil-based paint (usually consisting of three layers, whereas the American PPG paint has a single but thicker layer [20]). The direct coating cost per unit area using the new water-based coating was not significantly different from that of the regular oil-based paint, as shown in Table 3.

This new waterborne anticorrosion coating has been in service for five years and has shown excellent performance. It is evident that the anticorrosion lifespan of the new water-based coating was at least three times that of regular oil-based coatings. Moreover, only one construction and maintenance application every three years is required, allowing for applications on damp and rusted surfaces and significantly reducing construction process costs. Therefore, when calculating the total cost, the anticorrosion cost of the new water-based coating should be compared with that of regular oil-based coatings over a three-year period. A comparison of the equipment maintenance costs between the new water-based anticorrosion coating and regular oil-based coatings is presented in Table 4. The lifespan of the regular oil-based coatings was calculated to be one year, whereas the American PPG coating was assumed to have a two-year lifespan. Based on the 60,803 m^2^ corrosion protection area at Haimen Power Plant where the new coating has been applied for the last three years, the total anticorrosion cost for this area using the new water-based coating is approximately CNY 3.82 million. If traditional regular oil-based coatings were used, the estimated cost would be CNY 11.17 million. It can be seen that the new waterborne anticorrosion coating not only protects the environment and promotes the health of construction workers but also demonstrates significant economic benefits.

## 4. Conclusions

In this study, a modified titania waterborne coating was prepared and successfully applied to the steel structures of coastal power plants under subtropical marine climate. The main conclusions are summarized as follows:

(1) A waterborne methacryloxy silane-modified nanoscale titania coating was applied to a coastal power plant under subtropical marine climate. The coatings with 60,000 m^2^ exhibited no obvious failure after 5 years of field testing under extreme conditions.
(2) This waterborne coating could be coated under low-derusting grade conditions and even high humidity when it was used to repaint the steel structure.
(3) The methacryloxy silane coupling agent exhibited an activation index as high as- 97.5% when it was used to modify titania nanoparticles. The strength of the modified coatings was obviously improved through forming a grafted polymer by fluffy modified titania nanoparticles and resin molecules.

## Figures and Tables

**Table 1:** Sample number of different modified nanoscale titania.

Modified Nanoscale Titania	A1	A2	A3
Modified by	γ-amino propyl triethoxy silane	γ-(2,3-epoxy propoxy) propyl trimethoxy silane	γ-methacryloxy propyl trimethoxy silane

**Table 2:** Sample number of different titania waterborne anticorrosive coatings.

Waterborne Anticorrosive Coating	B1	B2	B3	B4
Titania types	A1	A2	A3	Unmodified

**Table 3:** Comparison of costs of different coatings.

Number	Coating Type	Price (CNY/kg)	Coating Thickness (μm)	Theoretical Coverage (m^2^)	Actual Cost per Unit Area
1	US PPG paint	170	230–250	5	68
2	Regular oil-based primer	50.385	40–50	6	31.24
	Regular oil-based intermediate paint	28.205	40–50	7	
	Polyurethane topcoat	41.667	40–50	8	
3	Waterborne anticorrosive coating	120.62	30–40	8	32.84

**Table 4:** Cost comparison of different coatings (based on three-year anticorrosion cost).

Number	Coating Type	Coating Cost (CNY/m^2^)	Labor Cost (CNY/m^2^)	Total Cost (CNY/m^2^)	Three-Year Anticorrosion Total Cost (Thousand CNY)
1	US PPG coating	68 × 1.5	30 × 1.5	147	8938
2	Regular oil-based coating	31.24 × 3	30 × 3	183.72	11,171
3	Waterborne anticorrosive coating	32.84	30	62.84	3821

## Data Availability

Data are contained within the article.
